# Prediction of protein binding sites in protein structures using hidden Markov support vector machine

**DOI:** 10.1186/1471-2105-10-381

**Published:** 2009-11-20

**Authors:** Bin Liu, Xiaolong Wang, Lei Lin, Buzhou Tang, Qiwen Dong, Xuan Wang

**Affiliations:** 1 Harbin Institute of Technology Shenzhen Graduate School, Shenzhen, PR China; 2 School of Computer Science and Technology, Harbin Institute of Technology, Harbin, PR China; 3 Department of Control Science and Engineering, Harbin Institute of Technology, Harbin, PR China; 4 School of Computer Science, Fudan University, Shanghai, PR China

## Abstract

**Background:**

Predicting the binding sites between two interacting proteins provides important clues to the function of a protein. Recent research on protein binding site prediction has been mainly based on widely known machine learning techniques, such as artificial neural networks, support vector machines, conditional random field, etc. However, the prediction performance is still too low to be used in practice. It is necessary to explore new algorithms, theories and features to further improve the performance.

**Results:**

In this study, we introduce a novel machine learning model hidden Markov support vector machine for protein binding site prediction. The model treats the protein binding site prediction as a sequential labelling task based on the maximum margin criterion. Common features derived from protein sequences and structures, including protein sequence profile and residue accessible surface area, are used to train hidden Markov support vector machine. When tested on six data sets, the method based on hidden Markov support vector machine shows better performance than some state-of-the-art methods, including artificial neural networks, support vector machines and conditional random field. Furthermore, its running time is several orders of magnitude shorter than that of the compared methods.

**Conclusion:**

The improved prediction performance and computational efficiency of the method based on hidden Markov support vector machine can be attributed to the following three factors. Firstly, the relation between labels of neighbouring residues is useful for protein binding site prediction. Secondly, the kernel trick is very advantageous to this field. Thirdly, the complexity of the training step for hidden Markov support vector machine is linear with the number of training samples by using the cutting-plane algorithm.

## Background

Identification of protein binding site has significant impact on understanding protein function. Development of fast and accurate computational methods for protein binding site prediction is helpful in identifying functionally important amino acid residues and facilitating experimental efforts to catalogue protein interactions. It also plays a key role in enhancing computational docking studies, drug design and functional annotation for the structurally determined proteins with unknown function [1].

**Figure 1:**
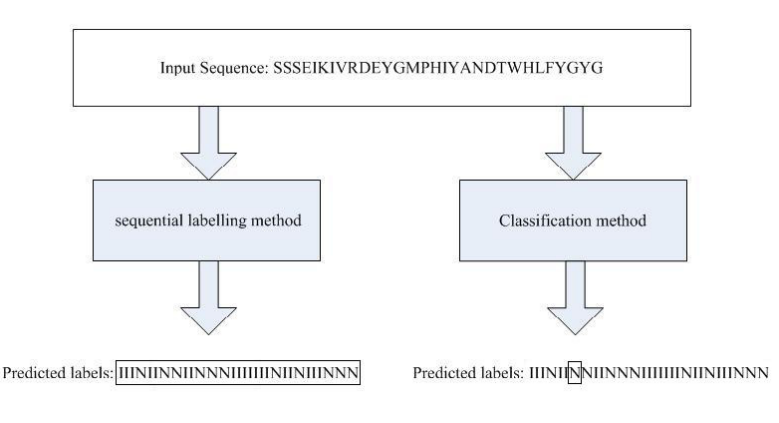
**An example of comparison of classification method with sequential labelling method for protein binding site prediction**. For the predicted labels, I and N represent interface residue and non-interface residue respectively.

Protein binding site prediction has been studied for decades [2-4]. Several machine learning methods have been applied in this field. These methods can be split into two groups: classification methods and sequential labelling methods. The essential difference between classification methods and sequential labelling methods is that the classification methods treat each residue as independent and ignore the extra information from neighbouring residues or constraints of a single sequence, i.e. treating the protein binding site prediction as a classification problem. In contrast, the sequential labelling methods are able to consider the correlations between labels, to include long-distance interaction and to model the protein sequence as a whole. An example of comparison of classification methods with sequential labelling methods for protein binding site prediction is shown in Figure 1.

Classification methods, such as support vector machines (SVM) [4-11] and artificial neural networks (ANN) [1,12-16], treat the protein binding site prediction as a classification task. These methods are based on the sequence or structure characteristics of known protein binding sites, such as sequence conservation [12], interface propensities [11,17], secondary structure [18], accessible surface area [1,5], 3D-motifs [19,20] and residue evolutionary information [7,9]. Because none of the individual property carries sufficient information that can be used to make an unambiguous identification of interface regions or patches, a combination of some of them (via either a linear combination [18] or machine learning [7,21]) is found to be effective in improving the accuracy of binding site prediction [22,23]. Kim et al. presented a hybrid approach by using both sequential and structural features [24]. Burgoyne and Jackson analyzed the ability of different key physicochemical attributes and binding surface properties, such as surface conservation, to predict the binding interface [25]. To improve prediction robustness and accuracy, meta-PPISP [26], a meta web server, combined results from different predictors including cons-PPISP [15], Promate [18] and PINUP [27]. Although the classification methods yield exciting results, these methods separately study the target residues and do not take the relation between two labels (interface or noninterface) of neighbouring residues into consideration. Some researchers noticed the importance of the inter-relation information between neighbouring residues for predicting protein binding sites and proposed several methods to use this information. Yan et al. [28] pointed out that interface residues tend to form clusters in the primary amino acid sequence and proposed a two-stage classifier. Chung et al. [10] used the clustering as a post-processing strategy to remove the isolated interface residues predicted by SVM. Although performance improvement is observed for these methods, they only use the local relation between neighbouring residues. In our previous study, we proposed a conditional random field (CRF)-based method [29], which treats the prediction of protein binding site as a sequential labelling task. In contrast to the traditional classification methods, such method uses the relation between neighbouring residues in a global fashion and shows better performance than traditional classification methods. Surveys of the methods for protein binding site prediction have been performed by two studies [30,31].

In this study, we introduce a novel machine learning scheme which overcomes several disadvantages associated with existing methods. The model is based on hidden Markov support vector machine (HM-SVM) [32], which treats the protein binding site prediction as a sequential labelling task based on the maximum margin criterion. Hidden Markov support vector machine (HM-SVM) is introduced initially for solving the problem of labelling sequence data that arises in the scientific fields such as bioinformatics and natural language processing. In addition to HM-SVM, some other methods also suit to label sequence, such as hidden Markov model (HMM) [33] and conditional random field (CRF) [34]. HMM is one of the most common methods for performing sequence labelling. HMM is able to model sequential dependencies by treating the label as a Markov chain, which avoids direct dependencies between subsequent observations and leads to an efficient dynamic programming formulation for inference and learning. It is a generative model that maximizes the joint probability distribution p(*x*, *y*), where *x *and *y *are random variables whose values take on all observation sequences and corresponding labelling sequences, respectively. Despite its success, HMM has at least three major limitations. 1) HMM is trained in a non-discriminative manner. 2) The conditional independence assumptions are too restrictive. 3) HMM is based on explicit feature representations and lacks the power of kernel-based methods. CRF is another successful method for sequence labelling, which is a discriminative probabilistic model. CRF uses a single exponential model for the conditional probability of all training labels, given the observation sequence. Therefore, the weight of an arbitrary feature can be learned from its global interactions with all the other features. This means that the weights of all the features within CRF can be traded off against each other. However, like HMM, CRF is based on explicit feature representations and lacks the power of kernel-based methods. HM-SVM follows the discriminative approach like CRF to model and comprises two additional crucial properties inherited from SVM: the maximum margin principle and a kernel-centric approach to learn non-linear discriminant functions. HM-SVM has been applied to some common problems in natural language processing, such as named entity recognition and part-of-speech tagging [32]. The experimental results are significantly better than those from other sequential labelling methods such as HMM and CRF. In this paper, three state-of-the-art methods including ANN, SVM and CRF are compared with our method for protein binding site prediction. These methods are trained by using common features derived from protein sequences and structures including protein sequence profile and residue accessible surface area. When tested on different types of data sets, the results show that our method performs well and its running time is several orders of magnitude shorter than that of the compared methods.

## Results

### Comparison with related methods

**Figure 2:**
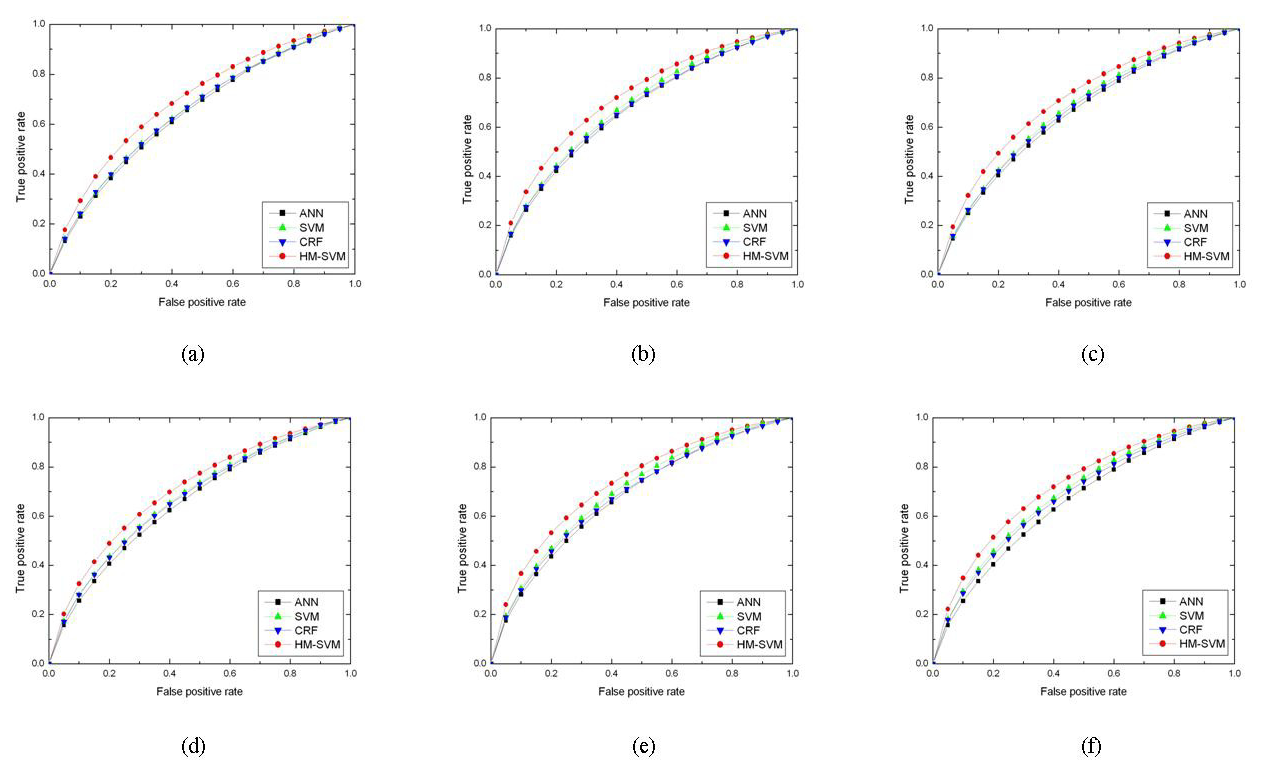
**ROC cures on the six data sets**. The ROC cures of ANN, SVM, CRF, HM-SVM on the six data sets: (a) Hetero-complex I, (b) Homo-complex I, (c) Mix I, (d) Hetero-complex II, (e) Homo-complex II, (f) Mix II.

Through the experiments reported here, the performance of the three following methods is compared with our method: artificial neural network (ANN), support vector machine (SVM) and conditional random field (CRF). These three methods are state-of-the-art methods within the field of protein binding site prediction [4,5,7,11,13,15,29,35]. ANN and SVM are classification methods, while CRF is a sequential labelling method. For detailed setup procedures of these methods please refer to the method section. Table 1 summarizes the performance of the various methods on the six data sets and the ROC cures are depicted in Figure 2. It is obvious that HM-SVM outperforms the other methods in terms of AUC for predicting protein binding sits. For each method, the performance on the homo-complex data sets is better than the performance on the hetero-complex data sets, which is consistent with previous study [15]. Generally speaking, the sequential labelling methods are consistently better than the classification methods. On the six data sets, HM-SVM yields the best performance according to MCC, F1 and AUC, which indicates that HM-SVM can obtain better trade-off between specificity^+ ^and sensitivity^+ ^automatically. Another sequential labelling method CRF gets the second best performance. The classification methods SVM and ANN are worst according to our experiment and SVM is better than ANN.

**Table 1:** Performance of HM-SVM versus other methods on all data sets

Data set	Method	Specificity^+ ^(random)^a^	Sensitivity^+ ^(random)^b^	F1	Accuracy	MCC	AUC	**Time (s)**^c^
Hetero-complex I^d^	ANN	37.6% (28.1%)	59.4% (16.7%)	46.0%	60.9%	18.9%	64.5%	326
	SVM	38.4% (28.1%)	59.8% (16.8%)	46.8%	61.8%	20.2%	65.4%	179461
	CRF	42.6% (28.1%)	55.2% (15.5%)	48.0%	66.5%	24.4%	65.3%	12151
	HM-SVM	44.9% (28.1%)	56.0% (15.7%)	49.8%	68.3%	27.4%	69.5%	356

Homo-complex I	ANN	39.0% (27.0%)	58.4% (15.8%)	46.6%	63.9%	22.1%	67.0%	586
	SVM	39.6% (27.0%)	61.9% (16.7%)	48.3%	64.2%	24.2%	68.6%	224979
	CRF	45.1% (27.0%)	59.2% (16.0%)	51.2%	69.5%	30.2%	67.6%	16961
	HM-SVM	45.4% (27.0%)	60.0% (16.2%)	51.7%	69.7%	30.9%	72.2%	588

Mix^e^I	ANN	40.3% (27.5%)	51.4% (14.1%)	44.7%	65.4%	20.8%	65.8%	1242
	SVM	39.5% (27.5%)	61.5% (16.9%)	48.1%	63.6%	23.3%	67.6%	831579
	CRF	44.3% (27.5%)	57.5% (15.8%)	49.9%	68.4%	28.0%	66.8%	28364
	HM-SVM	45.5% (27.5%)	58.0% (15.9%)	51.0%	69.4%	29.7%	71.2%	891

Hetero-complex II^f^	ANN	45.9% (34.9%)	60.5% (21.1%)	52.1%	61.3%	21.3%	65.8%	604
	SVM	47.9% (34.9%)	61.6% (21.5%)	53.9%	63.2%	24.6%	67.7%	160625
	CRF	51.6% (34.9%)	57.6% (20.1%)	54.3%	66.3%	28.0%	67.3%	13441
	HM-SVM	54.0% (34.9%)	56.7% (19.8%)	55.3%	68.0%	30.5%	70.7%	464

Homo-complex II	ANN	43.9% (32.3%)	66.7% (21.5%)	52.8%	61.5%	24.1%	68.1	856
	SVM	47.1% (32.3%)	63.1% (20.4%)	54.0%	65.2%	27.7%	70.2%	554054
	CRF	52.5% (32.3%)	59.7% (19.3%)	55.9%	69.5%	32.9%	68.7%	18124
	HM-SVM	53.3% (32.3%)	60.1% (19.4%)	56.5%	70.1%	34.0%	73.4%	851

Mix II	ANN	46.5% (33.3%)	53.4% (17.9%)	49.4%	63.7%	21.7%	65.8%	1260
	SVM	47.5% (33.3%)	62.3% (20.8%)	53.9%	64.5%	26.5%	69.2%	1316103
	CRF	52.2% (33.3%)	58.6% (19.5%)	55.2%	68.3%	30.9%	68.1%	856765
	HM-SVM	53.6% (33.3%)	58.6% (19.6%)	56.0%	69.3%	32.6%	72.4%	1320

Specificity^+ ^= TP/(TP+FP); Sensitivity^+ ^= TP/(TP+FN); F1 = 2 × Specificity^+ ^× Sensitivity^+^/(Specificity^+^+Sensitivity^+^); Accuracy = (TP+TN)/(TP+TN+FP+FN); MCC = (TP × TN-FP × FN)/; AUC: Area Under ROC Curve [61]. Where TP is the number of true positives (residues predicted to be interface residues that actually are interface residues); FP the number of false positives (residues predicted to be interface residues that are in fact not interface residues); TN the number of true negatives; FN the number of false negatives.

^a^Values in parentheses are randomly predicted values. The specificity^+ ^of random prediction is calculated as: the total number of interaction sites residues/the total number of residues.

^b^Values in parentheses are randomly predicted values. The sensitivity^+ ^of random prediction is calculated as: the total number of predicted residues as interaction sites by each method/the total number of residues.

^c^The total running time (second) for 5-fold cross-validation, including training and testing.

^d^Type I data set with minor interface as negative samples.

^e^The mixed data set of hetero-complexes and homo-complexes.

^f^Type II data set with minor interface as positive samples.

One important aspect of any protein binding site prediction method is its computational efficiency. We compare the running time of each method and the results are shown in Table 1. All the experiments are performed on a personal computer with CPU of Intel Pentium 2.2 GHz and memory of 3G. Concerning the computational time of different methods, only ANN is comparable with HM-SVM, but the prediction performance of ANN is considerably lower than that of HM-SVM. HM-SVM is two orders of magnitude faster than CRF and three orders of magnitude faster than SVM. Table 2 provides a qualitative estimation of computational costs of different methods.

**Table 2:** Summary of computational costs of different methods

	ANN	SVM	CRF	HM-SVM
Train	L	H	H	L
Test	L	H	L	L

L and H represent low computational cost and high computational cost, respectively.

### Influence of the number of training samples on the prediction performance and running time

**Figure 3:**
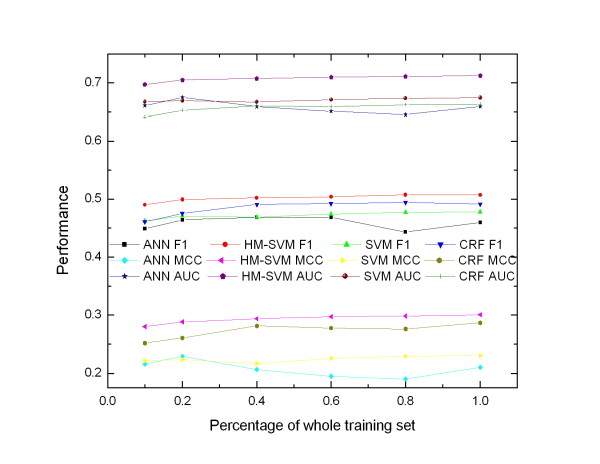
**Performance changing curves of different methods trained with different number of training samples on mix I data set**.

**Figure 4:**
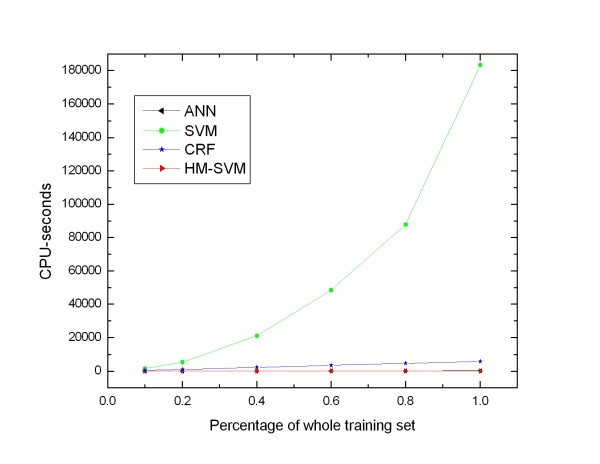
**Running time changing curves of different methods trained with different number of training samples on mix I data set**. The results are obtained on a personal computer with CPU of Intel Pentium 2.2 GHz and memory of 3G.

In order to investigate the influence of the number of training samples on the prediction performance and running time for each method, we generate a series of training sets with different number of training samples. We randomly select about one fifth of the chains from mix I data set as the test set (with 223 chains and 46345 residues), and the remaining chains are used as the training set (with 901 chains and 103084 residues). Different percentage of the whole training set is used as the training set. Figure 3 shows the performance of different methods trained with different number of training sample. For different training size, HM-SVM consistently outperforms the other methods in terms of F1, MCC and AUC. The curves of HM-SVM are smoother than those of the other methods, indicating that even trained with small number of training samples, HM-SVM can achieve stable performance. Figure 4 shows the relation between the running time and the number of training samples. The running time of the three methods ANN, CRF and HM-SVM approximately scales linearly with the number of training samples, while the running time of SVM increases significantly with the number of training samples from small to large, especially for large training set. Overall our method can be quickly trained on a large data set and get good results. Additionally, even trained on small data sets, our method can achieve stable performance.

### The inter-relation information between neighbouring residues is relevant for discrimination

To examine whether the inter-relation information between neighbouring residues learned by HM-SVM is relevant for discrimination, we run a control experiment. In the experiment, each residue is taken as an observation sequence in order to remove the relationship between residues of a protein sequence in the original training set. HM-SVM is trained on this modified training set and then tested on the original test set. The results on the six data sets are shown in Table 3. Compared with the results obtained on the original data sets, the results of HM-SVM on the modified data sets are lower in terms of F1-measure, MCC and AUC. Therefore, we conclude that the inter-relation information between neighbouring residues learned by HM-SVM is relevant for discrimination.

**Table 3:** Performance of HM-SVM trained on modified training sets

Data set	Specificity^+^	Sensitivity^+^	F1	Accuracy	MCC	AUC
Hetero-complex I	43.8%	58.3%	49.5%	66.7%	26.5%	69.1%
Homo-complex I	44.9%	58.4%	49.9%	68.4%	29.0%	71.9%
Mix I	42.8%	62.0%	49.6%	65.7%	27.2%	70.5%
Hetero-complex II	53.7%	56.1%	54.2%	67.4%	29.4%	70.3%
Homo-complex II	53.7%	55.4%	53.0%	69.0%	31.3%	72.4%
Mix II	54.5%	54.9%	54.5%	69.6%	31.8%	72.0%

### The window size has not significant influence on the performance

**Figure 5:**
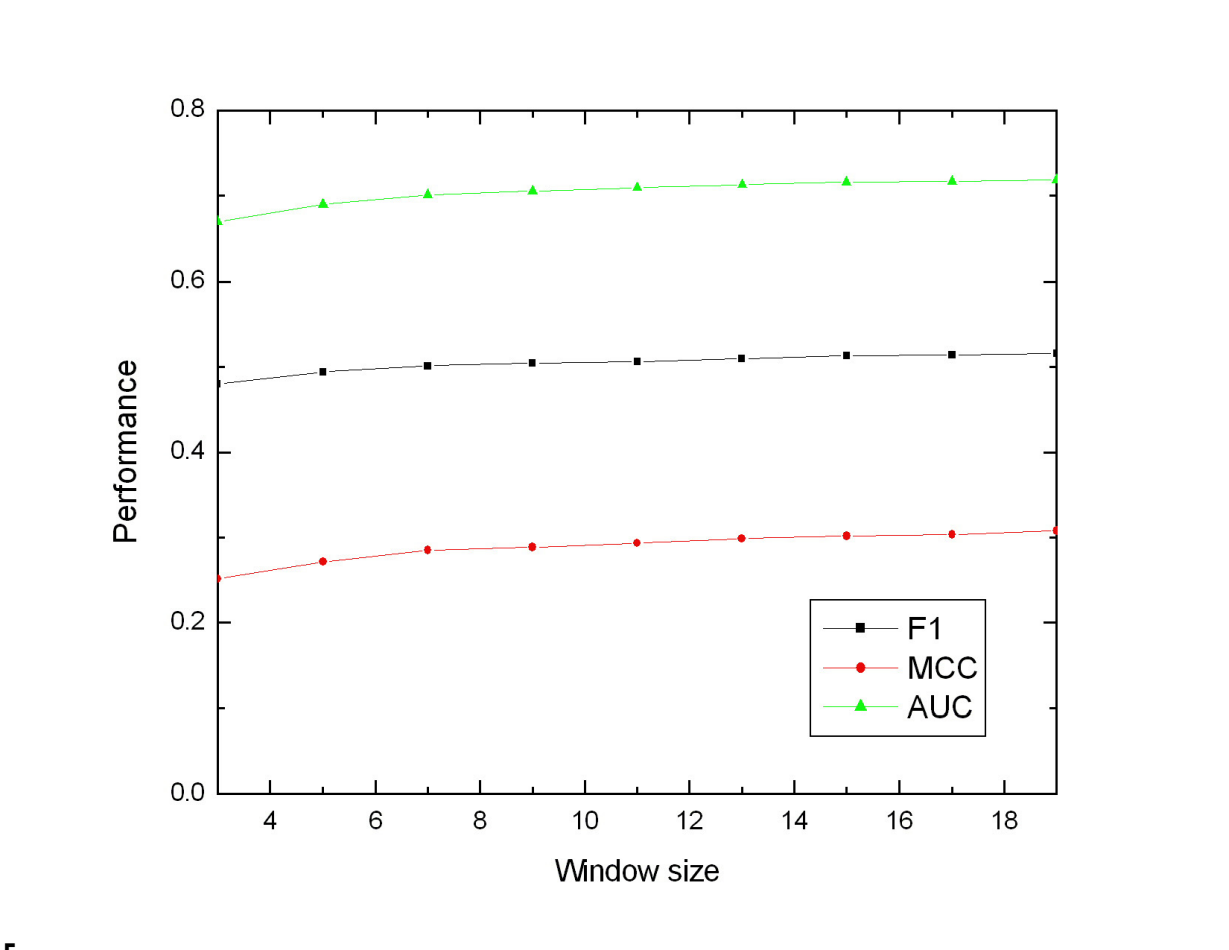
**Performance changing curves of HM-SVM using different window size on mix I data set**.

For each labelled residue, its profile feature and ASA feature are taken from a window containing *n *(odd number) nearest spatially neighbour residues (including the labelled residue). The window size is selected using embedded 5-fold cross-validation independently for each test set of the 5-fold cross-validation procedure. The influences of the window size on the performance of HM-SVM on mix I data set are illustrated in Figure 5. The results show that the window size has not significant influence on the performance of HM-SVM. When the window size is larger than 9, HM-SVM can achieve stable performance. The window size of 13 is used in this study, since it is the optimal window size for the SVM-based methods suggested by two related studies [10,11].

### Evaluation of the performance in the context of three-dimensional structures

**Figure 6:**
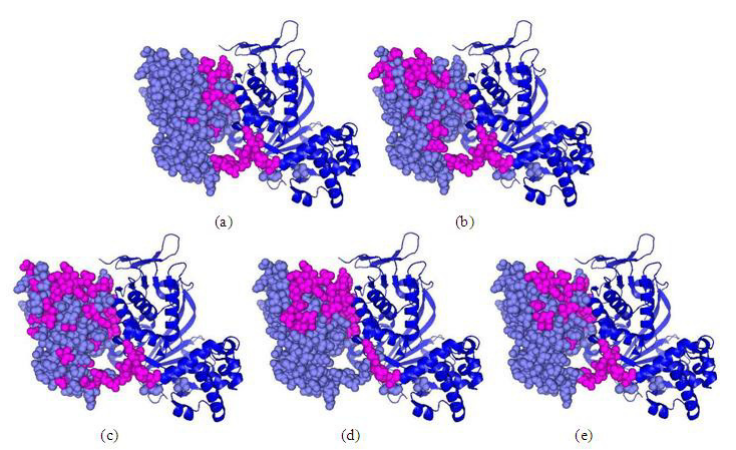
**Representative prediction results on hetero-complex I data set**. The target protein (PDB code 1ukv:Y) for which the predictions are made is shown in slate. Predicted interface residues are shown in magenta. The binding partner (PDB code 1ukv:G) is shown in blue. (a) The actual interface residues. (b) ANN. (c) SVM. (d) CRF. (e) HM-SVM.

**Figure 7:**
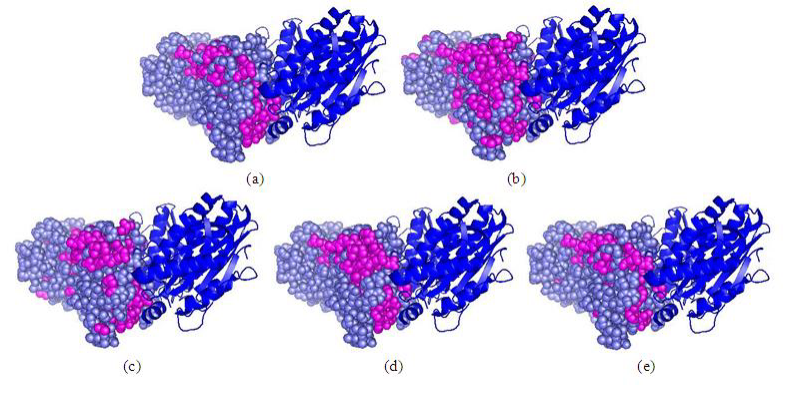
**Representative prediction results on homo-complex I data set**. The target protein (PDB code 1vz8:B) for which the predictions are made is shown in slate. Predicted interface residues are shown in magenta. The binding partner (PDB code 1vz8:A) is shown in blue. (a) The actual interface residues. (b) ANN. (c) SVM. (d) CRF. (e) HM-SVM.

To further evaluate the performance of our method, we examine predictions in the context of the three-dimensional structures. Two proteins are selected from hetero-complex I data set and homo-complex I data set and the results are shown in Figure 6 and Figure 7, respectively. As can be seen from Figure 6, most of the false positives predicted by the classification methods ANN and SVM locate on far away from the actual interface, while the false positives predicted by sequential labelling methods CRF and HM-SVM are roughly around the actual interface, especially HM-SVM can successfully distinguish interface and non-interface residues for this protein. This result is not surprising. Traditional classification methods separately study each residue without using the relation between two labels (interface or non-interface) of neighbouring residues. In contrast, sequential labelling methods take inter-relation information between neighbouring residues into consideration. Similar results are also observed for homo-complex. As shown in Figure 7, the prediction results of sequential labelling methods are better than those of classification methods. Among the four methods HM-SVM achieves the best prediction performance.

## Discussion

Methods which predict interface residues using amino acid sequence along with the structure of the target protein (but not the structure of the complex it forms with other proteins) are of interest, because relatively few experimentally determined structures of protein-protein complexes are currently available [28]. In this paper, a novel approach based on HM-SVM is used to label surface residues as interface residues or non-interface residues. This method is especially useful in the case where the structure of the target protein is known but the structure of the complex formed by it with one or more proteins is unknown. Our method does not only make full use of the relation between two labels (interface or non-interface) of neighbouring residues like CRF-based method, but also shares the advantages of the SVM-based method. Upon validation with six types of data sets, the method yields better results and its running time is several orders of magnitude shorter than that of the compared methods. Three factors contribute to the results. Firstly, the relation between labels of neighbouring residues is useful for protein binding site prediction. Secondly, the kernel trick is very advantageous to this field. Thirdly, the complexity of the training step for hidden Markov support vector machine is linear with the number of training samples by using the cutting-plane algorithm.

Two points should be emphasized in evaluating the significance of the protein binding site prediction results. Firstly, as shown in Table 1, for each method the performance on the data sets with minor interface as positive samples (Type II) is better than the performance on the data sets with minor interface as negative samples (Type I). The reason is that some of the false positives predicted on Type I data sets are in fact binding sites on Type II data sets. Although we consider all the known partners in the PDB file of a target protein on Type II data sets, some residues identified as false positives in performance measure of our method and the compared methods could in fact be residues that actually participate in contacts with proteins other than their known partners in the PDB file. Secondly, it should be noted that the data sets have highly unequal numbers of positive samples and negative samples. As noted by Yan et al. [28], in such a scenario, Matthews correlation coefficient (MCC) is a much better indicator of the performance of a method than accuracy, since accuracy favours the majority class. For example, if 80% of the residues are non-interaction residues, a predictor which always predicts a residue to be a non-interaction residue will achieve an accuracy of 80%. However, such a predictor is useless for protein binding site prediction. In addition, F1 is another good indicator, since it is a trade-off between specificity^+ ^and sensitivity^+^.

Recently, Šikić et al. [23] developed a classification method based on random forests for protein binding site prediction. Tested on a hetero-complex data set, the best results of their method are obtained when using a combination of sequence and 3D structure information. The method can achieve a specificity^+ ^(precision) of 76.45%, a sensitivity^+ ^(recall) of 38.06, an F1 of 50.82% and an accuracy of 80.05%. Although performance comparison between our method and their method is rather difficult owing to the different definition of interface residue and different data set, our method seems to show better performance on our hetero-complex II data set in terms of F1 (55.3%). Although their method shows higher accuracy, it should be noted that this performance measure is not a good indicator as discussed above. Therefore, we have reason to believe that the overall performance of our method is better than or at least comparable with theirs.

Our goal is to evaluate different machine learning methods and to show our method can effectively improve protein binding site prediction. Although our discussion focuses on a single protein binding site prediction system using two basic features (i.e. protein sequence profile and residue accessible surface area), other features can be added to our system to improve the performance. Therefore, researchers who are interested in finding novel characteristic features of protein binding sites could use our system to validate the effectiveness of their features and our system would also benefit from these features.

## Conclusion

Successful application of HM-SVM to protein binding site prediction is of great significance. There are many problems in the biology domain that can be formulated as sequential labelling tasks, such as protein disorder prediction [36], protein secondary structure prediction [37], kinase-specific phosphorylation site prediction [38], DNA binding site prediction [39], RNA binding site prediction [40], Prediction of cis/trans isomerization [41], protease substrate site prediction [42], disulfide connectivity prediction [43,44], functional residue prediction [45] and catalytic residue prediction [46]. Most of them are considered as challenging problems. Thus, these important sequential labelling tasks are potential areas for applications of HM-SVM.

## Methods

### Data sets

Complexes are selected from the Protein Data Bank (PDB) [47] and filtered by a number of stringent steps. All proteins with multi-chains, non-NMR structures and resolution better than 4 Å are selected. Two chains in a protein are defined as interacting pairs if more than one non-hydrogen atoms in each chain are separated by no more than 5 Å [8,15]. For PDB structures with more than two chains, each chain is selected for at most one time. The protein chains of <40 residues are discarded. The PQS web-server [48] is used to eliminate crystal packing complexes rather than biologically functional multimers. In order to get nonredundant protein chains, we perform clustering analysis to remove redundant chains. The NCBI BLASTClust program [49] is applied to the chains, with identity threshold of 25% (-S 25), minimal length coverage of 90% (-L 0.9). If two chains fall into different clusters, they should have pairwise sequence identity <25%. Thus, one representative chain of each cluster is selected. Finally, a total number of 1124 chains are obtained. By using sequence comparisons, the complexes are classified as homo-complexes or hetero-complexes. Two interacting protein chains are considered as homo-complexes, if over 90% of them are aligned and the sequence identity over the aligned region is more than 95% [15]; otherwise they are classified as hetero-complexes. Finally, 504 hetero chains and 620 homo chains are obtained. In a real application scenario, the complex type of a protein is often unknown. Therefore, different methods are also tested on the mixed data set of hetero-complexes and homo-complexes. Because of the low level of sequence identity, the resulting data sets are more challenging than the ones used in previous studies by our group [29] as well as by other authors [28].

A residue is considered to be a surface residue if its accessible surface area (ASA) of at least one of its atom is >2 Å^2 ^[50]. A surface residue is defined as interface residue if its ASA is decreased by more than 1 Å^2 ^upon complexation [27]. The ASA is computed using the DSSP program [51]. Since a protein chain within a complex with more than one chain may form more than one interface. Within these interfaces, there is generally a main large interface while residues in other minor interfaces can be treated as interface residues or non-interface residues [5,12]. In this study, we consider all the two cases and generate six data sets. The six data sets are available at additional file 1, 2, 3, 4, 5 and 6, respectively. Their statistical information is tabulated in Table 4.

**Table 4:** Summary of six types of data sets

Data set	Chains	**Res**.	**Surface res**.	Interface res.^a^
Hetero-complex I	504	109829	92797	26085 (28.1%)
Homo-complex I	620	172917	141295	38170 (27.0%)
Mix I	1124	282746	234092	64255 (27.4%)
Hetero-complex II	504	109829	92797	32386 (34.9%)
Homo-complex II	620	172917	141295	45633 (32.3%)
Mix II	1124	282746	234092	78019 (33.3%)

^a^Given in the bracket are the fraction of interface residues in the total number of surface residues.

Sequential continuous residue sequence segments are collected. Each residue within the segment is labelled as interface or non-interface residue. The segments are used to train and test HM-SVM.

### Hidden Markov support vector machine

Hidden Markov support vector machine (HM-SVM) is proposed by Altum et al. [32] for labelling sequence data. HM-SVM is a discriminative learning technique for labelling sequences based on a combination of the two most successful machine learning algorithms: support vector machine (SVM) and hidden Markov model (HMM). HM-SVM addresses all of the shortcomings of HMM, while retaining some of the key advantages of HMM, namely the Markov chain dependency structure between labels and an efficient dynamic programming formulation. Both HM-SVM and CRF adopt a discriminative approach to model and can account for overlapping features (labels can depend directly on features of past or future observations). In addition, HM-SVM comprises two additional crucial properties inherited from SVM: the maximum margin principle and a kernel-centric approach to learning non-linear discriminant functions.

Given an observed input sequence *x *= (*x*_1_, x_2_,..., *x*_*t*_), HM-SVM predicts a labelling sequence *y *= (*y*_1_, *y*_2_,..., *y*_*t*_) according to the following linear discriminant function:(1)

where, *k *is the order of hidden Moarkov model. *e*_*j*_(*x*, *y*_*i*_) is a emission feature function of the label at position *i *and the observation sequence. *t*_*j*_(*x, y*_*i*-*j*_,..., *y*_*i*_) is a transition feature function of the whole observation sequence and the labels between position *i *and *i*-*j *in the label sequence. The index *j *in *e*_*j *_and *t*_*j *_is feature serial number to represent different features. Emission weight vector *w*_*ej *_and transition weight vector *w*_*tj *_correspond with feature *e*_*j *_and *t*_*j*_, respectively. *w*_*ej *_is learned from each different *k*th-order labeling sequence *y*_*i*-*k*_... *y*_*i *_and *w*_*tj *_is learned from the adjacent labels.

Given a training set with *m *samples *S *= {(*x*^*n*^, *y*^*n*^) ∈ X × Y|*n *= 1,..., *m *drawn from a fixed distribution D_*X *× *Y*_, HM-SVM solves the following quadratic optimization problem during training:(2)

where *C *is a parameter that trades off margin size and training error. *ε*_*x *_is a slack variable. *l*(*y', y*) is Hamming loss function, which computes per-label loss for each individual label in *y'*, i.e.(3)

where *i *is the *i*th element of the label vector, and *L *is the length of the label vector.

Because the above quadratic optimization problem has exponentially many constraints, the cutting-plane algorithm is used to solve this problem up to a precision of *ε *in polynomial time [52,53]. In particular, the one-slack reformulation of the training problems is solved by using cutting-plane algorithm [54], which makes the complexity of the training step for HM-SVM linear in the number of training samples. For more details about HM-SVM and cutting-plane algorithm please refer to [32,54].

### Protein binding site prediction using HM-SVM

HM-SVM is used to label protein segments on protein surface. The segment is sequential continuous residue segment which are all surface residues. The label set is L = {I, N}, where I indicates the interface residue and N indicates the non-interface residue. Given an observation segment *x *= (*x*_1_, *x*_2_,..., *x*_*t*_), the label sequence *y *= (*y*_1_, *y*_2_,..., *y*_*t*_) (*y*_*i *_∈ L) with the highest score calculated by formula (1) is obtained by using HM-SVM. In this study, SVM^hmm ^toolkit version 3.10 [55] is used as the implementation of HM-SVM. We adopt the second-order Markov HM-SVM with linear kernel. The parameters *c *and *e *are set to 1 and 0.1 respectively, since they can get the best performance. Other parameters are set by default.

HM-SVM contains two types of features, emission feature and transition feature. Two kinds of features including spatially neighbouring residues profile and accessible surface area (ASA) which are common input features used by many studies [5,10,14,29] are taken as the emission features for HM-SVM. For each labelled residue, its profile features and ASA features are taken from the 13 nearest spatially neighbour residues (including the labelled residue).

Spatially neighbouring residue profile feature is taken from the Position-Specific Score Matrix (PSSM) outputted by PSI-BLAST [56]. PSI-BLAST [56] searches against the nrdb90 database from EBI [57] with parameters j = 10 and e = 0.001. The profile value *x *is scaled to [0,1] by using the following function [58]:(4)

The spatially neighboring residue profile feature is defined for each label-amino pair (y ∈ L and aa ∈ amino acid alphabet) as:(5)

where *PSSM*(*x*_*k*_, *aa*) is the element of position-specific scoring matrix for amino acid *aa *at position *k *in protein sequence. *x*_*k *_is from the spatially neighbouring residues list of *x*_*i*_.

Spatially neighbouring residue accessible surface area (ASA) features are computed using the DSSP program [51] and scaled by the nominal maximum area of each residue.(6)

where *x*_*k *_is from the spatially neighbouring residues list of residue *x*_*i*_.

Transition feature is defined for each label pair (*y*, *y'*) (y and y' ∈ L) as follows:(7)

where, *y*_*i*-1 _and *y*_*i *_are labels of residues at positions *i*-1 and *i *in the protein sequence *x*, respectively.

### Setup of compared methods

Three methods including artificial neural network (ANN), support vector machine (SVM) and conditional random field (CRF) are compared with our method. The setup procedures of these methods are briefly described as follows.

For ANN, Neural Network Toolbox in matlab is used as the ANN implementation and a feed-forward back-propagation neural network is used. The input features are linearly combined into an input layer with 21 × 13 nodes, which performs a nonlinear transform. There is a hidden layer with 20 nodes, whose output data are again fed to a final output layer with two nodes. The weights of the linear combinations in forming input to nodes are optimized on the training set to minimize the difference between predicted output value (ranging from 0 to 1) and the value coding the actual state (1 for I and 0 for N, where I indicates the interface residue and N indicates the non-interface residue).

SVM is probably the most widely applied protein binding site prediction method [4]. The input features are nonlinearly mapped to a feature space, in which a hyper-plane is obtained that optimally separates the data points corresponding to the I state from those corresponding to the N state. LIBSVM [59] is used as the SVM implementation with radial basis function as kernel. The values of γ and regularization parameter C are set to be 0.1 and 10, respectively.

FlexCRFs [60] is used as the CRF implementation. Because FlexCRFs cannot deal with continuous real value features, we modify it to solve this problem. The parameters of FlexCRFs take the optimal values provided by the authors (order = 1, init_lambda_val = 0.05) [29].

### Cross-validation

5-fold cross-validation is used to measure the performance of each method. The whole data set is randomly divided into five subgroups with approximately equal number of chains. The chain IDs of each subset for 5-fold cross-validation are available at additional file 7, 8 and 9, respectively. Each method is trained and tested five times with five different training and test sets. For each time, four subsets are used as training data and the remaining one is used as test data. The data sets have highly unequal numbers of positive samples and negative samples. If all surface residues are used in the training set, a method would be biased to predict a residue as a surface residue [11]. To obtain a balanced training set, we randomly remove a number of surface residues to make the ratio of positive and negative samples about 1:1.

## Authors' contributions

BL carried out the protein binding site prediction study, participated in designing the study, coding the experiment, drafting the manuscript and performing the statistical analysis. LL, BT, QD and XW participated in the design of the study and performed the statistical analysis. XLW conceived of the study, and participated in its design and coordination. All authors read and approved the final manuscript.

## Supplementary Material

Additional file 1 Hetero-complex I data set is available here. Click here for file

Additional file 2 Homo-complex I data set is available here. Click here for file

Additional file 3 Mix I data set is available here. Click here for file

Additional file 4 Hetero-complex II data set is available here. Click here for file

Additional file 5 Homo-complex II data set is available here. Click here for file

Additional file 6 Mix II data set is available here. Click here for file

Additional file 7 The chain IDs of each subset for 5-fold cross-validation on hetero-complex data set are available here. Click here for file

Additional file 8 The chain IDs of each subset for 5-fold cross-validation on homo-complex data set are available here. Click here for file

Additional file 9 The chain IDs of each subset for 5-fold cross-validation on mix data set are available here. Click here for file
